# Beneficial effect of kidney transplantation from a deceased donor on severe chronic refractory intradialytic hypotension – a case report

**DOI:** 10.1186/s12882-017-0662-y

**Published:** 2017-07-20

**Authors:** Ewa Ignacak, Dominik Cieniawski, Alina Bętkowska-Prokop, Czesław Osuch, Marek Kuźniewski, Władysław Sułowicz

**Affiliations:** 1 Department of Nephrology, Jagiellonian University, ul. Kopernika 15C, 31-501, Krakow, Poland; 2 First Department of Surgery, Jagiellonian University, ul. Kopernika 40, 31-501, Krakow, Poland

**Keywords:** Intradialytic hypotension, Kidney transplantation, Hemodialysis, Case report

## Abstract

**Background:**

Chronic refractory hypotension (IDH, intradialytic hypotension) is a rare but serious problem encountered in patients on hemodialysis. Patients with chronic hypotension are often disqualified by transplant teams from renal transplantation. This is due to the possibility of an enormous risk of ischemic complications.

**Case presentation:**

We describe a 44-year old female patient with severe refractory hypotension (mean BP 60/30 mmHg, the lowest 48/28 mmHg), which appeared after bilateral laparoscopic nephrectomy of the infected kidneys. The kidney transplantation from a deceased donor, with infusion of the two pressor amines (dopamine, dobutamine) was performed without technical complications and the blood pressure measurements were 100–120/70–80 mmHg. The immunosuppression regimen was tacrolimus (TAC) + mycophenolate mophetil (MMF) and steroids (GS). Pressor amines were discontinued on the 18th day after the transplantation. Because of delayed graft function, 4 hemodialysis treatments were performed. The patient was discharged from the hospital on the 22nd day with good function of the transplanted kidney (the concentration of serum creatinine 117 μmol/l). During one-year follow-up, the patient has been remaining stable with a very good graft function (serum creatinine 84 μmol/l) and normal blood pressure (115/70 mmHg).

**Conclusions:**

Proper preparation and adequate perioperative treatment allowed for safely performing kidney transplantation in the patient with severe IDH.

## Background

Dialysis hypotension (IDH, intradialytic hypotension) is a frequent complication in patients undergoing chronic hemodialysis (HD). It is estimated that it affects approximately 10–70% of patients. Significant differences in the incidence are associated with various definitions of this disease. KDOQI (The National Kidney Foundation’s Kidney Disease Outcomes Quality Initiative) defines IDH as a decrease in systolic blood pressure > 20 mmHg or a decrease in mean arterial blood pressure (BP) of > 10 mmHg with associated clinical symptoms (abdominal discomfort, nausea, vomiting, fainting, dizziness, muscle cramps, etc.). According to the KDOQI definition, IDH applies to approximately 20–30% of dialysis session [1].

IDH may occur as a form of acute (episodic), recurrent (for a minimum of 50% of the dialysis session) or rarely chronic dialysis hypotension, which is associated with the occurrence of BP <100 mmHg between HD sessions in 5–10% of patients. IDH is more common in elderly patients with cardiovascular disease, on long-term dialysis, in patients with diabetes, low levels of albumin, a high body mass index (BMI) in women, as well as in patients with low blood pressure before dialysis. The clinical consequences of IDH include primarily a deterioration in the quality of life and increased mortality due to cardiovascular complications. The effects of IDH are also associated with a lack of obtaining adequate dialysis, which is associated with the shortening of the dialysis session. The problems involve a vascular accesses - frequent clotting of arteriovenous fistulas [2, 3].

The occurrence of IDH is related primarily to the lack of an adequate volume of circulating blood during the dialysis session, which on the one hand results from insufficient refilling plasma, and the other – an impaired hemodynamic response from the myocardium and blood vessels.

Factors affecting the presence of IDH are dependent on the patient: being on antihypertensive medications, the presence of cardiovascular diseases, a considerable weight gain between dialyses, as well as on the manner of HD application (the concentration of sodium, calcium, the osmolarity of the dialyzate, the temperature of the dialysis liquid and the type of buffer used in dialysis), and on additional factors, such as anemia, hypoxia or intercurrent infections.

To date, the pathophysiology of IDH has not been completely understood. On the one hand, it is suggested that a reduced response of the cardiovascular system to vasopressor agents is associated with the occurrence of a reduced response to stimuli from the adrenergic receptors - there are indeed elevated levels of norepinephrine, but with a reduced density of alpha2 adrenergic receptors. In addition, there is a reduced response of alpha1 adrenergic receptors. Long-term dialysis treatment is also associated with an impaired baroreceptor function. Also reduced density and a response from the beta1 and beta2 adrenergic receptors have a clinical value. Another factor associated with the occurrence of IDH is an increased production of vasodilators (adrenomedullins and nitric oxide) as a result of an increased production of proinflammatory cytokines. Repeated episodes of organ hypoperfusion, the effect of IDH, are associated on the one hand with hypertrophy and myocardial fibrosis, and on the other - with an increase in the level of endotoxins, which in turn leads to an increased production of proinflammatory cytokines, oxidative stress and dysfunction of endothelial cells [2, 4]. Another cause of IDH may be bilateral nephrectomy leading to removal of sympathetic stimulus from native kidneys [5].

The occurrence of chronic IDH usually leads to disqualification of a patients from the kidney transplantation procedure. To date, there are few reports in the literature of a successful kidney transplantation with an improved BP control in patients with chronic IDH.

## Case presentation

A 44-year-old female patient with severe intradialytic hypotension, (the mean BP were 60/30 mmHg, the lowest BP 48/28 mmHg) was qualified for renal transplantation by a team from the Department of Nephrology, University Hospital after an in-depth diagnostic management.

The cause of the patient’s end-stage renal failure was rapidly progressive glomerulonephritis with anti-GBM treated in the past by plasmapheresis and Solu-Medrol pulses. In addition, since the age of 20 years, she had bilateral nephrolithiasis with recurrent urinary tract infections and hypertension. The patient was on hemodialysis for three years. One year after the commencement of dialysis, the patient underwent bilateral laparoscopic nephrectomy due to bilateral nephrolithiasis and recurrent urinary tract infections - which are an obstacle in qualifying patient for renal transplantation. Since that time, the patient has been severely hypotensive. She denied fainting, reported a significant weakness. In the course of hypotension, ischemic neuropathy with loss of vision in the right eye occurred. In addition, eye pain worsened during dialysis. Collaboration with the patient while renal replacement therapy was carried out was good, and her weight gain between dialysis sessions did not exceed 2000 ml. HD sessions were carried out in accordance with the standards for the IDH (the lowered temperature of the dialysis fluid, profiled sodium concentration, short-term infusion of colloids with declined BP). Additionally, the adrenal CT (computed tomography) scan was normal, as well as cortisol and ACTH (adrenocorticotropic hormone) levels. Histological examination of the rectal mucosa sample excluded amyloidosis. The results of cardiac studies (echocardiogram and electrocardiography) were also normal. The orthostatic stress test and Ewing’s battery tests showed no abnormalities of the autonomic nervous system, the heart rate variability was normal during the all the tests. Treatment with mineralocorticoids and midodrin did not result in the improvement of BP, but after the dopamine infusion, her blood pressure increased to satisfactory values (120/60 mmHg). The results of most relevant laboratory and diagnostic tests performed in patient are showed in Table 1. The patient was referred for renal transplantation and placed on the waiting list. The waiting time for kidney transplantation was 6 months.

**Table 1:** Results of most relevant laboratory and diagnostic tests

Type and name of diagnostic test	Result/Value	Normal laboratory range
Thyroid stimulating hormone	1.960	0.27–4.2
(TSH) [μIU/ml]		
Triiodothyronine (FT3) [pmol/l]	3.4	3.1–6.8
Cortizol [μg/dl]	11.7	2.3–19.4
Adrenocorticotropic hormone	26.3	8.0–58.0
(ACTH) [pg/ml]		
Hemoglobin (Hgb) [g/dl]	9.4	11.0–15.0
Total Cholesterol (TC) [mmol/l]	4.1	3.2–5.2
Sodium (Na) [mmol/l]	138	136–145
Echocardiography	Ejection Fraction 70%, mitral and tricuspid trace regurgitation	*not applicable*
Abdomen and Adrenal tomography	No pathologies within the abdomen and adrenal	*not applicable*
Ewing Battery tests	No abnormalities of the autonomic nervous system	*not applicable*
ECG and ECG stress test	Correct results	*not applicable*
Consultations:		
Neurological	No contraindications to kidney transplantation. No exact cause from intradialytic hypotension.	*not applicable*
Cardiac		
Endocrine		

**Fig. 1:**
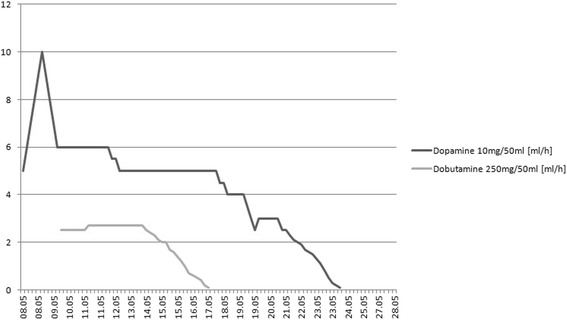
Pressing amines doses during hospitalization

**Fig. 2:**
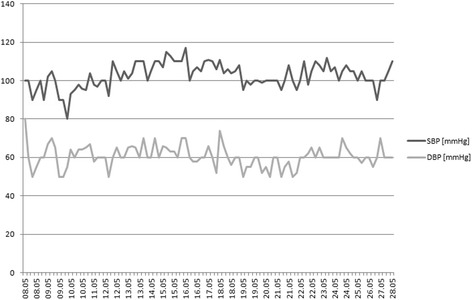
Blood pressure values during hospitalization

**Fig. 3:**
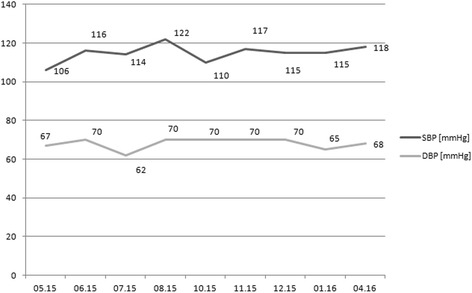
Long-term post-kidney transplantation blood pressure values

The kidney for transplantation was harvested from a deceased donor (33 years old male with overweight, after intracranial injury, without arterial hypertension and other diseases). The number of non-conforming HLA 2 A, 1 B and 1 DR, PRA max and the last was 0. The total time of ischemia was 22 h, and warm ischemia - 50 min. The patient used the following immunosuppression regimen: TAC + MMF + GS. Immediately prior to the transplantation procedure, her BP was 60/40 mmHg. Before the surgery, dopamine infusion at a dose of 5.3–7.4 μg/kg/min resulted in an increase of BP to 100–120 mmHg. The kidney transplantation procedure was performed without complications. In the second day after surgery, the patient was transferred to the Department of Nephrology. Due to the low value of BP < 100 mmHg, dopamine infusion and additionally dobutamine infusion at a dose of 2.4–3.6 μg/kg/min was ordered (Figs. 1 and 2). Initially, heart rate value was in range between 100 and 120 per minute, and during the reduction of aminopressor doses it decreased to normal values (80/min.). A delayed function of the transplanted kidney was observed, so 4 HD sessions were performed. During the post-operative period, the dosage of pressor amines was gradually reduced and stopped 18 days after transplantation. The patient was discharged from the hospital on day 22nd after transplantation in a good condition, with BP of 105/60 mmHg, the level of serum creatinine 117 μmol/L and eGFR 46 ml/min. One year after transplantation procedure, the graft function is stable, with the level of serum creatinine of 80 μmol/l and eGFR >60 ml/min, BP values ​​are approx. 105–120/60–70 mmHg (Fig. 3). The patient was well rehabilitated and returned to work.

## Discussion

In the described case, before qualification for renal transplantation, we excluded causes of IDH associated with diseases of the cardiovascular system, as well as causes of endocrine and autonomic nervous system dysfunction. Young age and lack of comorbidities also spoke in favor of the patient. Dialysis time before the kidney transplantation spanned 3 years. Undoubtedly, a factor that had an impact on the occurrence of hypotension in our patient was the performance of bilateral nephrectomy as preparation for renal transplantation. Double-sided nephrectomy or percutaneous ablation of renal sympathetic nerve are associated with a reduction in hyperactivity of the sympathetic system [5]. This has important clinical significance in patients with hypertension, particularly refractory and poorly controlled. An improvement in BP control and reduction in the number of antihypertensive medications after bilateral nephrectomy have been reported [6]. There are also reports of reduction in left ventricular mass and its diastolic dysfunction in the group of such patients [7]. In our patient, before bilateral laparoscopic nephrectomy, the BP values were normal. Reducing the activity of the sympathetic nervous system associated with the procedure, could therefore contribute to the onset of hypotension. In patients with chronic kidney disease (CKD) and patients on dialysis, an increased activation of the sympathetic nervous system, increased renin secretion, and increased activity of the renin-angiotensin-aldosterone system after bilateral nephrectomy were observed. Also this mechanism of BP control in our patient has been weakened. Unfortunately, due to the lack of opportunities, we did not determine in our patient the blood levels of adrenaline, noradrenaline, renin and aldosterone during the period of eligibility for transplantation.

Short-term dialysis (several months) prior to the IDH, the lack of co-morbidities, such as diabetes, autonomic neuropathy, and the correct state of the cardiovascular system (echocardiography, electrocardiography) indicate a good hemodynamic response from the myocardium and peripheral vasculature in our patient.

Rogan et al., reported the beneficial effects of exercise (stationary cycling without load during the dialysis session) on BP in a patient with IDH after bilateral nephrectomy. The exercise, performed during dialysis for 8 weeks after renal transplantation, contributed to carrying out a successful kidney transplant. The stimulation of chemo- and mechanoreceptors during exercise improve BP control [8]. Our patient was also instructed as to the need to perform resistance exercises, but such exercises did not produce any major clinical effect in improving BP.

In some cases, the beneficial effect of IDH results from the use of alpha 1-adrenergic receptor agonist - midodrin. In our patient, we did not observe any beneficial effect of the use of this drug.

The correct answer to the test use of the pressor amines (dopamine) before surgery allowed for qualifying the patient for renal transplantation.

Kim et al. describe a case of kidney transplantation in a 2-year-old child with dialysis hypotension and autonomic neuropathy, which received a transplant from an adult deceased donor. To obtain adequate perfusion of the transplanted kidney, the authors tried to achieve a value of systolic BP approx. 100 mmHg during the perioperative period. Due to the ineffective use of dopamine infusion for this purpose, the authors successfully employed an alpha agonist (neosinefrine) for a short time, despite the risk of ischemia development and the risk of thrombosis [9]. In the case of our patient, dopamine infusions for a short period of time in conjunction with dobutamine were effective. We also observed a clear correlation between the possibility of diminishing the doses of pressor amines and improvement of the function of transplanted kidney.

A similar observation was demonstrated also by Taiwan authors [10]. They described a 59-year old hypertensive diabetic female who during 6 years on HD developed dialysis associated hypotension (in the range 80–100/50–60 mmHg). Blood pressure increased to the range 120–140/60–80 mmHg on day 1st post transplantation. In other study Muscroft et al. [11] assessed the improvement of BP after a successful kidney transplantation in eight patients with IDH. They were immune high-risk patients with the presence of DSA (Donor Specific Antibodies) and had previously used the appropriate desensitizing procedures. Only one of eight patients received a kidney from a deceased donor, the others received a kidney from living donors. It should be emphasized that only four patients of this group had hypotension <100 mmHg between HD sessions. The authors draw attention to the large percentage of complications during the pre-transplant period (central retinal vein thrombosis) and the treatment period (bleeding, intestinal ischemia). One patient who received a kidney from a deceased donor died from complications associated with the occurrence of ischemic bowel disease. In these patients, different pressor amines (including norepinephrine) were used before transplantation [11]. No one of described patients with hypotension had previous bilateral nephrectomy.

## Conclusions

To summarize, we for the first time described beneficial effect of kidney transplantation in severe hypotensive patients after bilateral nephrectomy. At the moment there are no adequate standards dealing with the qualifications and kidney transplantation in patients with chronic IDH. A successful kidney transplantation observed in our patient suggests careful valuation of all potential candidates for kidney transplantation, including patients with IDH. It is very important, especially for the young patients, on short-term dialysis and free of additional disease. The necessary condition, however, is the extensive diagnosis before the kidney transplantation. On the basis of this case, as a minimum, we suggest testing the cardiovascular system in accordance with the standards governing the qualifications and further endocrine studies (rhythm of cortisol, ACTH) and studies of the autonomic nervous system based on the Ewing’s battery tests to exclude disorders of the autonomic nervous system. In the case of disorders of this system, it can be expected that the patient would not respond to treatment with dopamine. The use of vasoconstrictive drugs, such as phenylephrine, or high doses of norepinephrine may be associated with an increased incidence of fatal complications in this group of patients.

## Abbreviation

ACTH: Adrenocorticotropic hormone
BMI: Body mass index
BP: Blood pressure
CKD: Chronic kidney disease
CT: Computed tomography
DBP: Diastolic blood pressure
DSA: Donor specific antibodies
eGFR: Estimated glomerular filtration rate
GS: Glicocorticosteroids
HD: Hemodialysis
HLA: Human leukocytes antigen
IDH: Intradialytic hypotension
KDOQI: the National Kidney Foundation’s Kidney Disease Outcomes Quality Initiative
MMF: Mycophenolate-mofetil
PRA: Panel reactive antibody
SBP: Systolic blood pressure
TAC: Tacrolimus.
